# OA13 The skin as a snapshot of systemic inflammation

**DOI:** 10.1093/rap/rkad070.013

**Published:** 2023-09-27

**Authors:** Thomas Khoo, Athena Chin, Jenny Walker

**Affiliations:** Salford Royal Hospital, Salford, United Kingdom; Flinders Medical Centre, Adelaide, Australia; University of Adelaide, Adelaide, Australia; Flinders Medical Centre, Adelaide, Australia; Central Adelaide Local Health Network, Adelaide, Australia; Flinders Medical Centre, Adelaide, Australia

## Abstract

**Introduction:**

This case focuses on a polymorphic rash of multidisciplinary interest in the setting of seemingly disparate multi-organ inflammatory phenomena. The case highlights the use and shortcomings of skin histopathology in diagnosing systemic conditions, particularly when faced with the dilemma of ordering the differential diagnoses of infection, inflammation and autoimmunity, and the subsequent decision-making around antibiotics or immunosuppression.

**Case description:**

A 71-year-old man was admitted to a tertiary referral hospital in Adelaide, Australia, with fever (39.4^o^C), malaise and vomiting following a brief fishing expedition. No sick contacts were noted, although six months prior he had hiked through mountainous Pennsylvania, USA.

Shortly after admission, he developed an acute, widespread rash involving his arms, legs, chest and abdomen with relative sparing of the back and face. The rash was erythematous, non-pruritic and polymorphic with papulonodular areas, some coalescing into plaque-like lesions. In areas on the forearms, there were vesicular and pustular components to the rash.

Multiple other inflammatory manifestations were subsequently determined: synovitis of the proximal interphalangeal joints, widespread lung nodulosis with associated ground-glass opacities, transverse colon colitis, bilateral epididymo-orchitis, vocal cord ulceration, and bilateral conjunctivitis.

Blood tests showed a normocytic anaemia (haemoglobin 82g/L; mean cell volume, MCV 90fL), neutrophilia (11x10^9/L) and elevated C-reactive protein (CRP, 350mg/L). No serological, culture or imaging evidence of infection was found on extensive investigations, including bronchoscopy, colonoscopy and echocardiogram. PET scan revealed no FDG-avid malignancy, vasculitis or infection. A skin biopsy showed both medium vessel and leukocytoclastic vasculitis.

Broad spectrum antibiotics appeared to result in partial improvement, with resolution of the rash, defervescence, improvement in ground-glass opacities on chest imaging and stabilisation of the CRP at 200mg/L. The anaemia, however, worsened (nadir haemoglobin 73g/L) and became symptomatic, requiring transfusion, and the patient developed marked hypoalbuminaemia (nadir 12g/L) with associated peripheral and pleural effusions.

In the context of the skin biopsy results, a provisional diagnosis of systemic medium-vessel vasculitis was made, and steroid therapy commenced with significant subjective improvement, improvement in anaemia and hypoalbuminaemia, and normalisation of CRP.

Two weeks after discharge, genetic testing returned with a somatic variant of the UBA1 gene (Met41Leu), revising the diagnosis to VEXAS (Vacuoles, E1 Enzyme, X-linked, Autoinflammatory, Somatic Syndrome).

**Discussion:**

Since VEXAS was first described in 2020, there have been case reports of patients with this diagnosis, typically older males, who present with protean inflammatory manifestations. As a disease entity which exists on a novel continuum of autoinflammation and myelodysplasia, VEXAS can mimic other conditions including vasculitis, inflammatory arthritis, Sweet’s syndrome and relapsing polychondritis. Indeed, there exists a subset of patients previously labelled with these diagnoses who have subsequently been found to have mutation of the UBA1 gene.

We report a case of VEXAS initially diagnosed as a medium-vessel vasculitis on the basis of the only definitive finding, namely the result of a skin biopsy, following extensive investigation for infection. Although biopsy remains an important investigation in diagnosing vasculitis, this case demonstrates that there are now instances where genetic testing supersedes histopathology in obtaining a diagnosis. In this case, testing for a UBA1 variant was prompted by widespread inflammatory phenomena and persistent anaemia. Notably, however, VEXAS has almost universally been reported with macrocytosis but the patient in this case had a persistent normocytic anaemia.

This case features some distractors: the reported sudden onset of symptoms, recent travel overseas and a partial response to antibiotic therapy with resolution of the patient’s fevers and widespread skin rash. In retrospect, with the diagnosis of VEXAS in mind, it is likely that this improvement represented disease fluctuation rather than response to antibiotics.

Outstanding questions from this case include which, if any, of the organs involved in this patient’s initial presentation most suggested VEXAS. Whether a bone marrow biopsy in the work-up of unexplained systemic inflammation would have obtained an earlier diagnosis with the finding of vacuoles (noting that these are not universally present in patients with VEXAS) is also uncertain. Finally, the lack of macrocytosis is unexplained and atypical for VEXAS.

**Key learning points:**

VEXAS is thought to be underdiagnosed and should be considered as a differential diagnosis, particularly in an older male patient who presents with widespread inflammatory features and haematological abnormalities.

VEXAS can cause a variety of rashes and skin histopathology demonstrating vasculitis may reflect systemic inflammation rather than systemic vasculitis.

In the frequently encountered quandary of infection versus inflammation/autoimmunity, a seeming response to antibiotics does not necessarily confirm an infectious aetiology and may have other explanations such as the anti-inflammatory effects of antimicrobials (e.g. doxycycline) or natural fluctuations of inflammatory disease activity.

